# Galectin-2 Agglutinates *Helicobacter pylori* via Lipopolysaccharide Containing H Type I Under Weakly Acidic Conditions

**DOI:** 10.3390/ijms25168725

**Published:** 2024-08-10

**Authors:** Takaharu Sasaki, Midori Oyama, Mao Kubota, Yasunori Isshiki, Tomoharu Takeuchi, Toru Tanaka, Takashi Tanikawa, Mayumi Tamura, Yoichiro Arata, Tomomi Hatanaka

**Affiliations:** 1 Faculty of Pharmacy and Pharmaceutical Sciences, Josai University, 1-1 Keyakidai, Sakado, Saitama 350-0295, Japan; 0814ssk@gmail.com (T.S.); oyamami@josai.ac.jp (M.O.); traum.4869@icloud.com (M.K.); isshiki@josai.ac.jp (Y.I.); takeuchi@dpc.agu.ac.jp (T.T.); tanakato@josai.ac.jp (T.T.); tanikawa@josai.ac.jp (T.T.); 2 School of Pharmacy, Aichi Gakuin University, 1-100 Kusumoto-cho, Chikusa-ku, Nagoya, Aichi 464-8650, Japan; 3 Faculty of Pharmaceutical Sciences, Teikyo University, 2–11–1 Kaga, Itabashi-ku, Tokyo 173-8605, Japan; m-tamura@pharm.teikyo-u.ac.jp (M.T.); arata@pharm.teikyo-u.ac.jp (Y.A.); 4 School of Medicine, Tokai University, 143 Shimokasuya, Isehara, Kanagawa 259-1193, Japan

**Keywords:** galectin-2, galectin-3, *Helicobacter pylori*, lipopolysaccharide, stomach, Lewis antigen, pH, lectin

## Abstract

Galectins are β-galactoside-binding animal lectins involved in various biological functions, such as host defense. Galectin-2 and -3 are members of the galectin family that are expressed in the stomach, including the gastric mucosa and surface mucous cells. Galectin-3 exhibits aggregation and bactericidal activity against *Helicobacter pylori* in a β-galactoside-dependent manner. We previously reported that galectin-2 has the same activity under neutral pH conditions. In this study, the *H. pylori* aggregation activity of galectin-2 was examined under weakly acidic conditions, in which *H. pylori* survived. Galectin-2 agglutinated *H. pylori* even at pH 6.0, but not at pH 5.0, correlating with its structural stability, as determined using circular dichroism. Additionally, galectin-2 binding to the lipopolysaccharide (LPS) of *H. pylori* cultured under weakly acidic conditions was investigated using affinity chromatography and Western blotting. Galectin-2 could bind to *H. pylori* LPS containing H type I, a Lewis antigen, in a β-galactoside-dependent manner. In contrast, galectin-3 was structurally more stable than galectin-2 under acidic conditions and bound to *H. pylori* LPS containing H type I and Lewis X. In conclusion, galectin-2 and -3 might function cooperatively in the defense against *H. pylori* in the stomach under different pH conditions.

## 1. Introduction

Galectins are a class of animal lectins with an affinity for β-galactosides. They are expressed in a variety of animal species, including sponges, nematodes, amphibians, and mammals. Sixteen galectins have been identified and are expressed in various organs and tissues in mammals [1,2,3]. Galectins are classified into three types—proto-, chimera-, and tandem-repeat-type galectins—based on their protein structure. The proto- and chimera-type galectins have a single carbohydrate-recognition domain (CRD), whereas the tandem repeat type has two CRDs per molecule [2,4]. Proto- and chimera-type galectins can noncovalently form dimers or oligomers and crosslink complex glycoconjugates by binding to β-galactoside structures. By binding to host cell surface glycoconjugates, they affect a variety of cells, including fibroblasts, keratinocytes, macrophages, T cells, B cells, and mucous cells, and are involved in important biological processes, such as cell adhesion, leukocyte migration, angiogenesis, host defense, and wound healing [5,6]. Galectins can also bind to cell surface glycoconjugates on pathogenic microorganisms and play a role in the infection process; for example, galectin-4 and -8 bind to certain *Escherichia coli* strains expressing human blood group antigens and kill them [7].

Galectin-2 (Gal-2), a prototype galectin, forms dimers. It is evolutionarily conserved, with 67% amino acid homology between rat Gal-2 (rGal-2) and human Gal-2 (hGal-2) [8]. Gal-2 is mainly expressed in the digestive tract in mice [9] and also in humans, as indicated in the human protein atlas database “https://www.proteinatlas.org/ (accessed on 24 August 2023)” [10]. Gal-2 expression has been reported in surface mucous cells and mucous neck cells in the stomachs of mice and rats [8,11,12]. rGal-2 has been suggested to be involved in the barrier function of gastric mucus via crosslinking with mucin, a component of gastric mucus, in vitro [13]. The expression of Gal-2 is reduced in patients with gastric cancer and lymph node metastasis [14]. In addition, the infection of mice with *Helicobacter felis*, another bacterium of the genus *Helicobacter*, decreases Gal-2 expression in the stomach at both the mRNA and protein levels [15]. These findings suggest that Gal-2 plays a role in defense against *H. pylori* infection of the stomach. In a previous study, we found Gal-2 expression in the gastric mucosa and showed that it exhibited β-galactoside-dependent aggregation and bactericidal activities against *H. pylori* under neutral conditions [16]. However, the aggregation effect of Gal-2 under acidic conditions remains unclear, especially with regard to the site of its action in the stomach and the types of ligands involved.

The interior of the stomach is strongly acidic because of the gastric acid secreted by parietal cells, which defends against pathogen infection. To protect against gastric acid, surface mucous cells secrete mucus and bicarbonate ions into the stomach. Thus, the pH of the stomach varies from strongly acidic on the luminal side to weakly acidic and neutral on the surface of gastric mucous cells [17,18,19]. *Helicobacter pylori*, a Gram-negative helical bacillus, causes gastric diseases, such as gastritis and peptic ulcers, and is found in the gastric mucosa, particularly within 25 µm of the surface [20]. *Helicobacter pylori* produces urease, which hydrolyzes urea into ammonia and carbon dioxide. This enzyme neutralizes the acid in the stomach to enable the survival, colonization, and infection of bacteria [21]. Therefore, the aggregation effect of Gal-2 should be investigated under weakly acidic conditions under which *H. pylori* cells survive.

Gal-2 binds to the surface glycoconjugates of *H. pylori* in a β-galactoside-dependent manner [16]. The outer membrane of *H. pylori* contains lipopolysaccharide (LPS), a glycolipid similar to that in other typical Gram-negative bacteria. LPS is comprised of lipid A, core oligosaccharides, and *O*-antigen. *Helicobacter pylori* has a fucosylated *O*-antigen called the Lewis antigen, which differs from that in other typical Gram-negative bacteria [22]. Lewis antigens include H type I and Lewis X, which have a β-galactoside structure [22]. Therefore, these glycoconjugates can be recognized by Gal-2.

Several galectin subtypes are expressed in the stomach [8,11,12]. Among them, galectin-3 (Gal-3), which is a chimera-type galectin that can form dimers or oligomers, is present in the gastric mucosa and exhibits β-galactoside-dependent aggregation and bactericidal activities against *H. pylori* [11,23,24,25]. Gal-3 binds directly to *H. pylori* LPS *O*-antigen in an *O*-antigen-defective mutant strain [24]. However, the effects of its ligand glycoconjugates on *H. pylori* and its activity under slightly acidic conditions have not been investigated. In addition, a comparison of the underlying molecular mechanisms of *H. pylori* aggregation induced by the different galectin subtypes has not been reported.

In the present study, the aggregation effect of Gal-2 against *H. pylori* was assessed under weakly acidic conditions. The structural stability of Gal-2 was examined under various pH conditions using circular dichroism (CD) spectroscopy. Gal-2 binding to the LPS of *H. pylori* cultured under weakly acidic conditions was investigated using affinity chromatography and Western blotting. The structural stability and ligands of Gal-3 were also investigated. We also discuss the roles of Gal-2 and Gal-3 in the stomach during *H. pylori* infection.

## 2. Results

### 2.1. Gal-2 Agglutinates H. pylori at pH 6.0 but Not at pH 5.0

**Figure 1:**
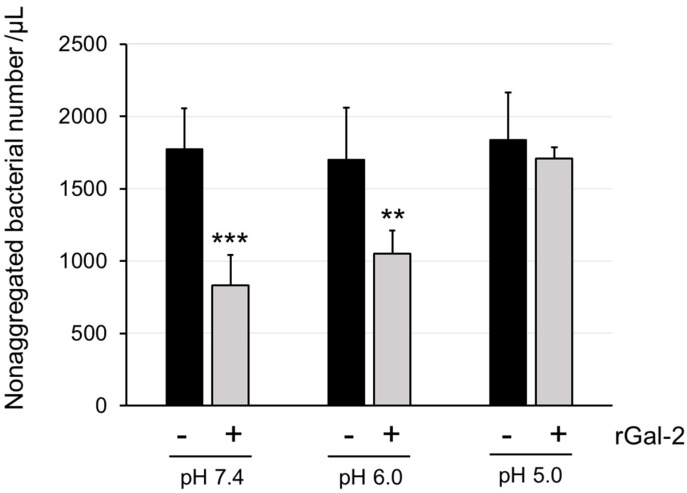
The aggregation of *Helicobacter pylori* induced by Gal-2 at various pH. The number of nonaggregated bacteria with (gray bar) or without (black bar) rGal-2 (93.9 µg/mL) under pH 7.4, 6.0, and 5.0 conditions is shown. Each bar represents the mean ± standard deviation (SD) from five samples. **, *p* < 0.01, ***, *p* < 0.001 by Student’s *t*-test (vs. without rGal-2).

To investigate the aggregation effects of Gal-2 on gastric mucus, an *H. pylori* suspension was mixed with rGal-2 solutions at pH 7.4, 6.0, and 5.0. Although Gal-2 agglutinated *H. pylori*, the size and shape of the aggregated *H. pylori* were heterogeneous. Therefore, the aggregation effect of Gal-2 was evaluated by calculating the number of nonaggregated *H. pylori* cells (Figure 1). rGal-2 reduced the number of nonaggregated *H. pylori* cells compared with that observed without rGal-2 at pH 6.0, as well as at pH 7.4. However, the aggregation effect of rGal-2 was not observed at pH 5.0.

### 2.2. Gal-2 Structure Is Altered at pH 5.0

**Figure 2:**
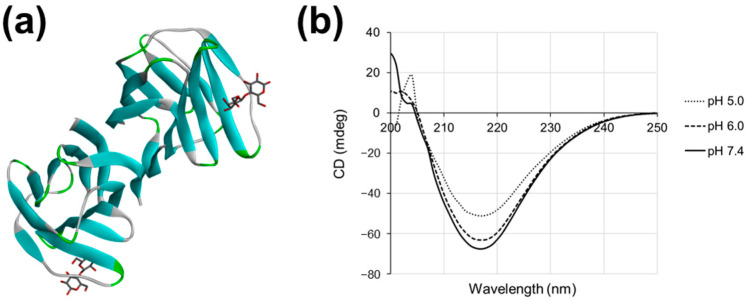
The structure and circular dichroism (CD) spectra of Gal-2 at various pH. (**a**) The structure of the hGal-2 dimer with lactose (PDB: 1HLC) shown using a ribbon diagram. The β-sheet is colored in blue. (**b**) CD spectra of rGal-2 (0.5 mg/mL) at pH 7.4, 6.0, and 5.0.

The reduction in the *H. pylori* aggregation activity of Gal-2 at pH 5.0 suggested a change in the structure of this galectin. The Gal-2 CRDs were characterized by their β-sheet-rich structures (Figure 2a; PDB: 1HLC). To confirm the structural changes in Gal-2, the CD spectra of rGal-2 at pH 7.4, 6.0, and 5.0 were compared. The negative peak at approximately 217 nm, which is derived from the β-sheet structure, was observed under all pH conditions (Figure 2b). The signal of the negative peak at pH 6.0 was slightly altered and further changed at pH 5.0 compared to that at pH 7.4. This suggests that the structure of Gal-2 is altered at pH 5.0.

### 2.3. Helicobacter pylori Contains LPS with Lewis Antigen

**Figure 3:**
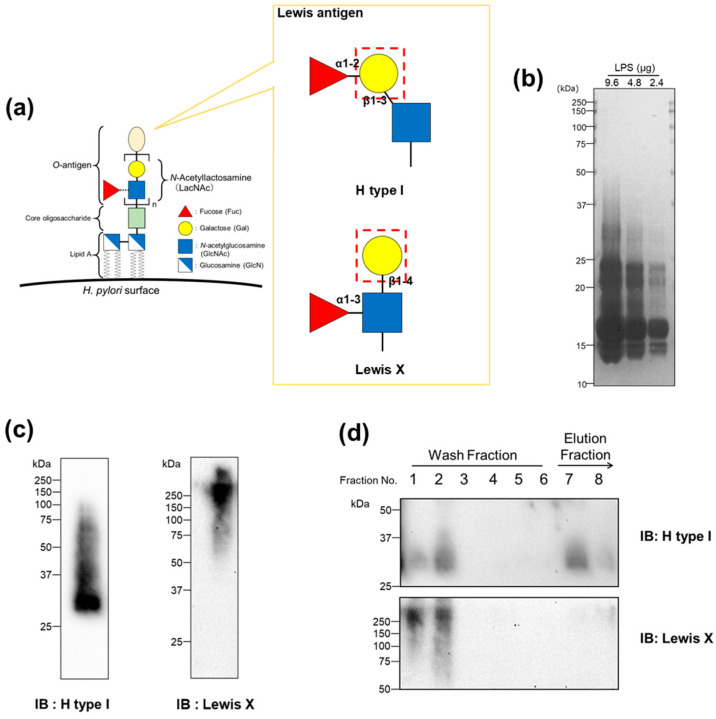
Lipopolysaccharide (LPS) from *Helicobacter pylori* contains a Lewis antigen. (**a**) A schematic representation of the Lewis antigen contained in *H. pylori* LPS and *N*-acetyllactosamine (LacNAc). The β-galactoside structures are surrounded by red dotted lines. (**b**) The silver staining of LPS extracted from *H. pylori* (ATCC43504) using the hot aqueous phenol extraction method. The lyophilized LPS extract was applied to the lanes of a sodium dodecyl sulfate–polyacrylamide gel and electrophoresed. The gel was silver-stained after treatment with periodic acid. (**c**) Western blotting of LPS extract from *H. pylori* using anti-lacto-*N*-fucopentaose I (H type I) and anti-SSEA1 (Lewis X) antibodies. (**d**) Western blotting of LPS extract from *H. pylori* after affinity chromatography using an hGal-2-immobilized column. After washing with phosphate-buffered saline containing 1 mM EDTA (E-PBS) (Fraction Nos. 1–6), the bound materials were specifically eluted with 0.1 M lactose (Fraction No. 7, 8).

Because *H. pylori* has Lewis antigens containing a β-galactoside structure, it is possible that Gal-2 binds to antigens, such as H type I and Lewis X (Figure 3a) [26,27]. To confirm the presence of LPS in the *H. pylori* extract, we electrophoresed the extract on a sodium dodecyl sulfate–polyacrylamide gel and silver-stained the gel. Ladder-like bands were detected on the gel (Figure 3b). The LPS composition was analyzed using gas–liquid chromatography after repurification (Table 1). Comparing the results of our LPS compositional analysis with those of previous reports [26,27], we found that, although the compositional ratios slightly differed, the sugars contained (fucose, galactose, *N*-acetylglucosamine, glucose, _D_-*glycero*-_D_-*manno*-Heptose, _L_-*glycero*-_D_-*manno*-Heptose) were the same across studies. We verified the presence of Lewis antigens in the extracted LPS. The LPS extracts were subjected to Western blotting using anti-H type I and Lewis X antibodies (Figure 3c). Both antigens were detected even when incubated at pH 6.0.

Next, we investigated whether Gal-2 binds to LPS via these glycans using affinity chromatography. The extracted LPS was applied to an hGal-2-immobilized column and washed (wash fraction). The bound materials were then specifically eluted with 0.1 M lactose, which possesses the β-galactoside structure (elution fraction). Each fraction was subjected to Western blotting using anti-H type I and anti-Lewis X antibodies (Figure 3d). H type I, but not Lewis X, was detected in the eluted fraction using Western blotting.

### 2.4. The Structure of Gal-3 Is Altered at pH 4.3 and Binds to H. pylori LPS Containing H Type I and Lewis X Structures

**Figure 4:**
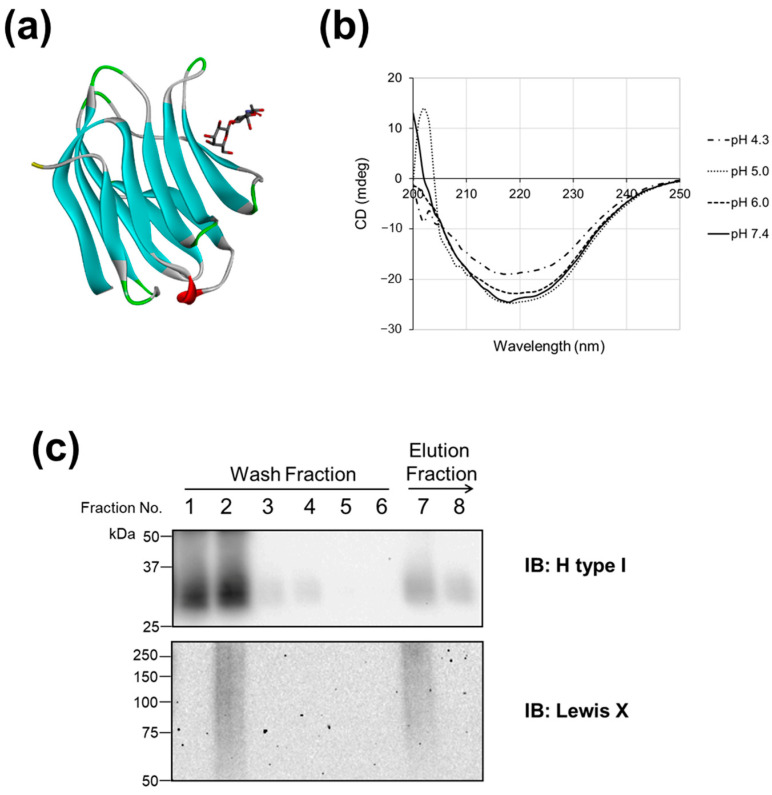
The structural stability of Gal-3 and its interacting Lewis antigens. (**a**) The structure of the galectin domain of hGal-3 with lactose (PDB: 1KJL) shown using a ribbon diagram. The β-sheet is colored in blue. (**b**) Circular dichroism (CD) spectra of hGal-3 (0.2 mg/mL) at various pH. (**c**) Western blotting of lipopolysaccharide (LPS) extract from *Helicobacter pylori* after affinity chromatography using an hGal-3-immobilized column. After washing with E-PBS (Fraction Nos. 1–6), the bound materials were specifically eluted with 0.1 M of lactose (Fraction Nos. 7, 8).

The structural stability of Gal-3, which also exhibits aggregation and bactericidal activity against *H. pylori*, in the gastric mucus environment has not been reported. Because the Gal-3 CRD is also characterized by β-sheet-rich structures (Figure 4a; PDB: 1KJL), the CD spectra under various pH conditions were evaluated (Figure 4b). The negative peak indicating the β-sheet structure at approximately 217 nm was observed under all pH conditions. The signal of the negative peak was almost unchanged at pH 7.4, 6.0, and 5.0 but changed at pH 4.3 (Figure 4b).

The Gal-3 ligand on *H. pylori* was investigated by affinity chromatography using an hGal-3-immobilized column (Figure 4c). H type I and Lewis X structures were detected in the eluted fraction using Western blotting. These results suggest that the reported aggregation activity of Gal-3 occurs through its binding to LPS containing H type I and Lewis X antigens in *H. pylori*.

## 3. Discussion

We previously reported the β-galactoside-dependent aggregation and bactericidal activities of Gal-2 against *H. pylori* [16]. In this study, the aggregation activity was investigated in more detail in terms of pH dependence, structural stability, ligands involved, and comparison with Gal-3.

In the previous study, Gal-2 was shown to agglutinate *H. pylori* under neutral conditions, as culturing *H. pylori* and extracting LPS were technically difficult under weakly acidic conditions at that time. However, the interior of the stomach is strongly acidic due to the gastric acid secreted by parietal cells and weakly acidic around the surface mucous cells, which secrete mucus and bicarbonate ions [17,18,19]. Gal-2 is present in the gastric mucosa [16]. Therefore, the *H. pylori* aggregation effect of Gal-2 under acidic conditions should be investigated. The aggregation effect was maintained under neutral conditions to pH 6.0 and was lost at pH 5.0 (Figure 1). This was supported by the CD spectra results under various pH conditions (Figure 2): the CRD structure of Gal-2 could be changed at pH ≤ 5.0, which may weaken its sugar-binding ability. The β-galactoside-binding activity of Gal-1, which is structurally similar to Gal-2, also changes under acidic conditions [28]. His45 of Gal-1 is protonated at a pH below 5.7, resulting in the loosening of the CRD structure and reduced ligand-binding activity. Because the corresponding His residue is also conserved in the CRD of Gal-2, it is possible that a similar mechanism occurs in Gal-2. In addition, the CRD structure of Gal-2 may be more sensitive to extrinsic factors, such as reactive oxygen species [29]. Gal-2 agglutinates *H. pylori* in the gastric mucus immediately above or near the gastric surface mucous cells, although the detailed mechanisms remain undetermined.

Based on the CD spectrometry results, Gal-3 was structurally more stable than Gal-2 under acidic conditions (Figure 2 and Figure 4b). The ligand-binding ability of Gal-3 for a sugar probe A-Tetra (blood group antigen; GalNAcα1–3(Fucα1-2)Galβ1–4Glc) varies at pH from 7.5 to 4 [30]. The affinity of Gal-3 for the model ligand was reduced but was retained to a certain extent at pH 5.0 [30], whereas the conformation of Gal-3 was unaffected, as suggested by the CD spectrum (Figure 4b). The CD spectrum is reflective of the secondary structure of a protein but not of its tertiary or quaternary structures. Therefore, it is possible that the affinity of galectins for their ligands was affected, without significant changes in their CD spectra. The affinity between A-tetra and Gal-3 decreased slightly at pH 5.0 compared to that at pH 7.5, and the affinity was the lowest at pH 4.0 [30]. The dramatic change in affinity at pH 4.0 is apparently consistent with our CD spectrum results at pH 4.3. Because ligand-binding specificity often depends on the geometry of the binding site, hydrophobicity, and polarity (charge distribution), changes in electrostatic potential at the molecular surface of hGal-2 (PDB: 1HLC) and hGal-3 (PDB: 1KJL) at pH 5.0 and 7.0 were analyzed using the Poisson–Boltzmann server ver. 3.6.2 “https://server.poissonboltzmann.org/ (accessed on 19 July 2024)” (Supplementary Figure S1). The molecular surface near the CRD of Gal-2 was more negatively charged at pH 7.0 than at pH 5.0. On the other hand, the area near the CRD of Gal-3 was positively charged at pH 7.0 and remained positively charged at pH 5.0. This suggested that Gal-2 might be more sensitive to pH than Gal-3.

In our previous report, the aggregation activity of Gal-2 against *H. pylori* was inhibited by the addition of lactose, which indicates that Gal-2 interacts with a glycoconjugate with the β-galactoside structure. Lewis antigens, which attach to *H. pylori* LPS on its outer membrane, have a β-galactoside structure, which makes them possible Gal-2 ligands. The direct binding of Gal-2 to these saccharides has been shown using frontal affinity chromatography analysis employing fluorescently labeled oligosaccharides and recombinant galectin proteins [31]; Gal-2 could directly bind to LNFP-I (Fucα1-2Galβ1-3GlcNAcβ1-3Galβ1-4Glc) containing an H type I epitope (Fucα1-2Galβ1-3GlcNAc) but not to LNFP-III (Galβ1-4(Fucα1-3)GlcNAcβ1-3Galβ1-4Glc) containing a Lewis X epitope (Galβ1-4(Fucα1-3)GlcNAc). We found that Gal-2 also binds to *H. pylori* LPS containing saccharides using affinity chromatography; Gal-2 could bind to LPS containing the H type I but not to the Lewis X of *H. pylori* at pH 6.0 (Figure 3d). Our results are consistent with those of a direct binding study [31]. The structure of LPS from the *H. pylori* strain (ATCC 43504) used in this study has been reported [26,32]. Although the detailed structure of *H. pylori* LPS has not been confirmed, we used a general method to extract LPS and confirmed its presence and composition via silver staining and gas–liquid chromatography, respectively (Figure 3b, Table 1). Further detailed structural analysis of affinity-purified *H. pylori* LPS is required to clarify whether Gal-2 binds directly to the H type I epitope attached to LPS.

Gal-3 bound to *H. pylori* LPS containing H type I and Lewis X (Figure 4). These results are consistent with those of previously reported direct binding assays employing fluorescently labeled sugars [31] and oligosaccharides derived from *H. pylori* LPS [33]. The *O*-antigen of *H. pylori* is composed of several repeats of the *N*-acetyllactosamine (LacNAc) structure (Figure 3a), and a part of the repeated unit is estimated to be modified with fucose, which has H type I and Lewis X structures. Such internal structures can be bound by Gal-3 because it has a more extended pocket for carbohydrate binding than Gal-2. Thus, Gal-2 binds to the β-galactoside structure at the terminus of glycan, whereas Gal-3 binds not only to the terminus but also to its interior [34]. Therefore, Gal-3 binds to *H. pylori* LPS containing the Lewis antigen; however, it is possible that Gal-3 recognizes parts of the LPS oligosaccharide other than the H type I or Lewis X epitope. To prove this, further investigations, such as structural analysis of LPS oligosaccharides bound by Gal-3 and more detailed direct binding assays, are required.

**Figure 5:**
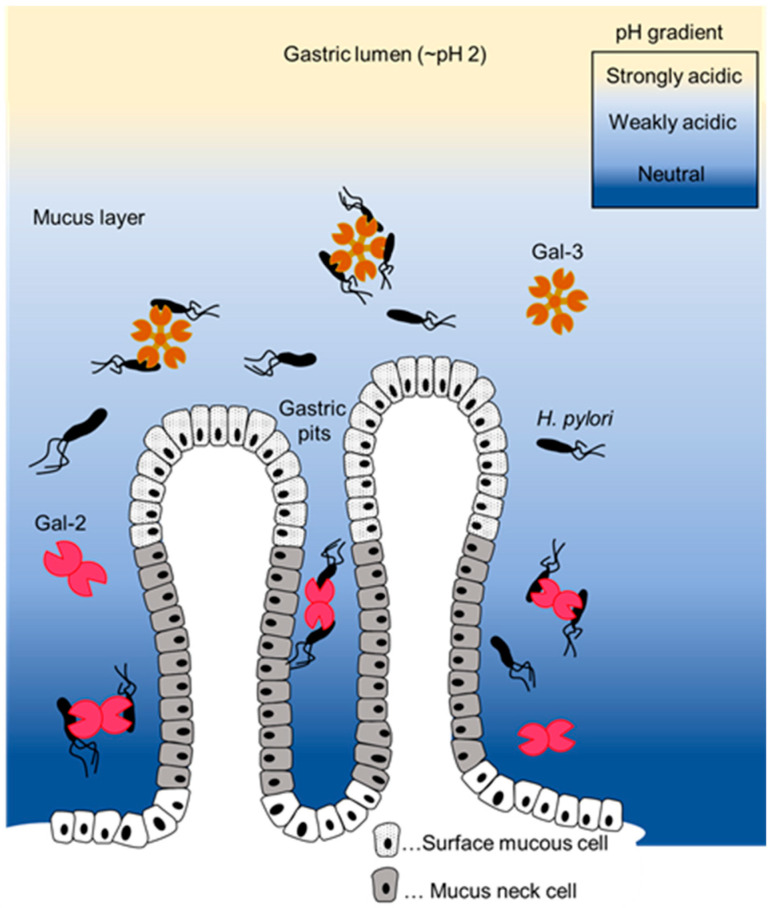
A schematic diagram of the potential biological defense mechanisms against *Helicobacter pylori* infection by Gal-2 and Gal-3 in the stomach. The aggregation effects of Gal-2 and Gal-3 against *H. pylori* in the stomach are shown.

The findings of the present study suggest that Gal-2 and Gal-3 have different roles and functions in their cooperative defense against *H. pylori*. Therefore, Gal-2 and Gal-3 might act as bactericidal agents against *H. pylori* at different sites. Gal-3 shows aggregation and bactericidal effects against *H. pylori* on the luminal side of the stomach. When *H. pylori* escapes the defense mounted by Gal-3, Gal-2 could agglutinate and kill bacteria near gastric surface mucous cells (Figure 5). A more detailed comparative study is required to confirm this hypothesis and to clarify the roles of Gal-2 and Gal-3 against *H. pylori* infection. With the increasing incidence of drug-resistant *H. pylori* infection [35,36,37], the development of novel therapeutic strategies is desired. Because Gal-2 expression is reduced by *Helicobacter* infection [15], a compound that can suppress this reduction or increase Gal-2 expression may have beneficial effects on *H. pylori* infection. Recently, galectin-mimicking molecules have been synthesized [38]. Recombinant Gal-2, Gal-3, their mimics, and compounds that regulate their expression are promising candidates for the development of anti-*H. pylori* drugs in the future.

This study had limitations. First, the detailed mechanism by which the CRD structure of Gal-2 was changed under acidic conditions has not been elucidated. Second, in vivo experiments are needed to confirm where Gal-2 aggregates *H. pylori* inside the stomach (e.g., near gastric surface mucus cells or in gastric mucus). β-Galactoside-independent mechanisms of the aggregation activity of Gal-2 against *H. pylori* may exist. Although these unresolved issues remain, the present study demonstrates that Gal-2 and Gal-3 might have distinct roles in *H. pylori* infection. These issues should be further examined in the future.

## 4. Materials and Methods

### 4.1. Preparation of Recombinant Gal-2 and -3 Proteins

Recombinant hGal-2, hGal-3, and rGal-2 were expressed in *E. coli* (BL21) and affinity-purified using an asialofetuin-immobilized Sepharose column, as described previously [16]. For the hGal-3-expressing plasmid, pET-hGal-3, an artificial gene encoding the full-length hGal-3 protein with optimized codon usage, was synthesized by Fasmac (Kanagawa, Japan) and subcloned into the NdeI and BamHI sites of the pET21a *E. coli* expression vector. For hGal-2 and rGal-2 expression plasmids, the pET-hGal-2 and pET-rGal-2 genes, respectively, corresponding to their open reading frames were subcloned into the NdeI and BamHI sites of the pET21a vector. *Escherichia. coli* cells transformed with these galectin expression plasmids were cultured, and protein expression was induced by adding isopropyl-β-thiogalactopyranoside. The cells were harvested and sonicated, and the debris was removed by centrifugation. The recombinant protein was affinity-purified using an asialofetuin-immobilized column. The collected fractions were separated by SDS-PAGE and stained using Coomassie G-250 stain (Bio-Rad, Hercules, CA, USA), and we confirmed the purity of the recombinant galectin proteins (Supplementary Figure S2): affinity-purified Gal-2 and Gal-3 gave approximately a single band in the elution fraction, although small amounts of degradation products of Gal-3 were also detected. Proteins in the eluted fractions that showed almost a single band were concentrated by ultrafiltration and exchanged with PBS (E-PBS) containing 1 mM EDTA at various pH values (pH 7.4, 6.0, and 5.0) using an Amicon Ultra-15 filter apparatus (Merck Millipore, Burlington, MA, USA). To remove LPS from the purified protein, Detoxi-Gel (Thermo Fisher Scientific, Fremont, CA, USA) was added to the protein solution, and the mixture was incubated at 4 °C for 30 min with mixing. The resin was removed using centrifugation at 2300× *g* at 4 °C for 1 min. The supernatant thus obtained was sterilized with a 0.2 µm filter and used for the subsequent experiments. The concentration of each purified protein was determined using a Bio-Rad Protein Assay (Bio-Rad), with bovine serum albumin (BSA) as a standard.

For structural analysis employing CD spectroscopy, the buffer of the recombinant protein solution was changed to 20 mM NaH_2_PO_4_, 150 mM NaCl, pH 6.0, 0.1 M CH_3_COOH, 150 mM NaCl, pH 5.0 or 0.1 M CH_3_COOH, and 150 mM NaCl, pH 4.3, using a Mini Dialysis kit (1 kDa cut-off; GE Healthcare, Chicago, IL, USA).

### 4.2. Helicobacter pylori Aggregation Assay

The *H. pylori* strain ATCC43504 was purchased from the American Type Culture Collection (ATCC, Manassas, VA, USA). The preparation of the *H. pylori* suspension and the aggregation assay were performed as described previously [16]. In brief, *H. pylori* colonies growing on a selective agar plate (Nissui Pharmaceutical, Tokyo, Japan) were harvested and suspended in Hank’s balanced salt solution (HBSS, Invitrogen, Carlsbad, CA, USA) at an optical density at 600 nm (OD_600_) of 1.0. Thereafter, 100 µL each of the *H. pylori* suspensions and recombinant protein solution were mixed and incubated for 1 h at 37 °C. The mixture was observed with a microscope, and the density of nonaggregated bacteria was calculated as follows: Number of bacteria/µL = Number of bacteria per unit constant area (0.2 mm × 0.2 mm) × 40.5.

### 4.3. Circular Dichroism Spectroscopy

The CD spectra of the recombinant rGal-2 protein dissolved in a buffer with pH 7.4, 6.0, or 5.0 at a concentration of 0.5 mg/mL were obtained using a Jasco J-720WI instrument with a Peltier temperature control unit (JASCO Corporation, Tokyo, Japan), as previously reported [29]. For recombinant hGal-3, the protein was dissolved in a buffer with pH 7.4, 6.0, 5.0, or 4.3.

### 4.4. Preparation of H. pylori Lipopolysaccharides

*Helicobacter pylori* colonies growing on selective agar plates (Nissui Pharmaceutical) were harvested and resuspended in physiological saline. The cells were then inoculated in a liquid medium (Brain Heart Infusion containing 0.1% glucose, 10% fetal bovine serum, pH 6.0), as described previously [39], and cultured at 37 °C, with shaking at 150 rpm, for 3 days under microaerobic conditions.

LPS extraction was performed as described previously [32,40] with slight modifications. Cultured *H. pylori* cells were harvested using centrifugation at 5000× *g* at 25 °C for 5 min and washed twice with PBS containing 0.15 mM CaCl_2_ and 0.5 mM MgCl_2_. The *H. pylori* pellet was suspended in 10 mL of PBS and disrupted by sonication. Proteinase K (QIAGEN, Hilden, Germany), at a final concentration of 20 µg/mL, was added to the solution, followed by incubation at 65 °C for 1 h. Thereafter, 40 units/mL of DNase I (Nippon gene, Tokyo, Japan) and 10 µg/mL of RNase A (Nippon gene) were added, and the mixture was incubated overnight at 37 °C, with shaking at 150 rpm. After incubation, the *H. pylori* extract was used for LPS extraction using the hot aqueous phenol extraction method [41]. In brief, equal volumes of the disrupted *H. pylori* extract and 90% (*v*/*v*) phenol were mixed and incubated at 70 °C for 15 min with periods of vigorous shaking. The aqueous layer was collected, and the phenol layer was re-extracted by adding an equal volume of H_2_O. To the pooled aqueous layer, sodium acetate was added at a final concentration of 0.5 M, and then 10 times the volume of 95% EtOH was added; the mixture was incubated overnight at −20 °C. LPS was collected by centrifugation at 8500× *g* at 4 °C for 30 min and suspended in H_2_O, followed by dialysis using a Mini Dialysis kit (GE Healthcare) to remove the residual phenol. The prepared LPS solution was lyophilized and stored as a powder.

### 4.5. Purification and Chemical Analysis of LPS

*Helicobacter pylori* LPS powder (10 mg) was dissolved in 10 mM Tris-HCl (pH 7.5) containing 5 mM MgCl_2_ at 2 mg/mL. Ten micrograms per milliliter DNase and 100 µg/mL RNase were added and incubated at 37 °C for 3 h, and then 50 µg/L of proteinase K was added. After incubation at 37 °C for 3 h, the solution was dialyzed (cellulose tubes, MwCO 14,000) against pure water. After centrifugation of the inner solution at 9000× *g* for 10 min at 20 °C, the supernatant was purified by repeated ultracentrifuged at 105,000 × *g* for 10–20 h. The precipitate was dissolved in 2 mL of water and centrifuged at 9000× *g* for 10 min at 20 °C. The supernatant was lyophilized as purified LPS [42].

Sugars were analyzed by gas–liquid chromatography (Shimadzu GC2014, Kyoto, Japan) equipped with a DB-210 capillary column (Agilent technologies, Santa Clara, CA, USA) using alditol acetate [27,42]. After the hydrolysis of purified LPS with 4 M trifluoroacetic acid at 100 °C for 3 h, 0.1 M HCl at 100 °C for 48 h, or 4 M HCl at 100 °C for 16 h, sugars were conventionally reduced with NaBH_4_ and then acetylated with acetic anhydride/pyridine to give an alditol acetate derivative.

For fatty acid analysis, purified LPS was hydrolyzed with 4 M HCl at 100 °C for 16 h. Lipids were extracted with 40% ether–hexane and converted into methyl ester derivatives with diazomethane. The derivatives were analyzed using a gas–liquid chromatograph (Shimadzu GC2014) equipped with a SPELCOWAX 10 capillary column (Sigma-Aldrich, St. Louis, MO, USA).

The total and inorganic phosphorus were determined using a colorimetric method (P-molybdate reaction) [43].

### 4.6. Affinity Chromatography

*Helicobacter pylori* LPS was dissolved in 1 mL of E-PBS at a concentration of 5 mg/mL. The LPS solution was applied to an immobilized hGal-2 or -3 column (bed volume 1.0 mL; 12.3 mg hGal-2/mL gel or 4.37 mg hGal-3/mL gel), prepared as described previously [44]. After washing the column with 5 mL of E-PBS, the absorbed materials bound to hGal-2 or -3 were eluted with 2 mL of E-PBS containing 0.1 M lactose. The volume fraction used throughout the experiment was 1 mL.

### 4.7. Silver Staining and Western Blotting

The LPS dissolved in H_2_O or affinity-purified fractions were mixed with 5× sodium dodecyl sulfate-polyacrylamide gel electrophoresis (SDS-PAGE) sample buffer (250 mM Tris-HCl, pH 6.8, 10% SDS, 50% glycerol, and a few milligrams of bromophenol blue) and boiled for 10 min. The samples were subjected to SDS-PAGE using an AE-7350 Compact PAGE System (ATTO, Tokyo, Japan). For silver staining, the gels were fixed in a fixing solution (EtOH–acetic acid–purified water = 4:1:5) for 90 min. The gels were soaked in periodate solution (EtOH–acetic acid–14% periodate solution–purified water = 4:0.5:0.5:5) and shaken. After washing with purified water, the gels were stained with an AE-1360 Ez Stain Silver kit (ATTO), according to the manufacturer’s instructions, and images were captured using a ChemiDoc XRS+ (Bio-Rad, Hercules, CA, USA). For Western blotting, the separated materials were transferred onto PVDF membranes (Bio-Rad) using a wet electroblotting system, Mini Trans-Blot Cell (Bio-Rad). Immunoblotting was performed using an iBind^TM^ Western Device (Life Technologies, Carlsbad, CA, USA), according to the manufacturer’s instructions, with appropriate antibodies. The blots were visualized with Luminata Crescendo (Merck Millipore), and signals were detected using a ChemiDoc XRS+ (Bio-Rad). The antibodies used were as follows: anti-lacto-*N*-fucopentaose I antibody (1:2000, Clone R-17F; Funakoshi, Tokyo, Japan) and HRP-conjugated anti-mouse IgG antibody (1:1000, Merck Millipore) for the detection of H type I; anti-SSEA 1 antibody (1:250, Clone MC-480; Novus Biologicals, Centennial, CO, USA); and HRP-conjugated anti-mouse IgM antibody (1:5000, Jackson ImmunoResearch, West Grove, PA, USA).

### 4.8. Statistics

Data are presented as the mean ± standard deviation (S.D.). Student’s *t*-test was used for comparisons between two groups. *p*-values < 0.01 or 0.001 were considered statistically significant.

## 5. Conclusions

Gal-2 agglutinated *H. pylori* under weakly acidic conditions. Gal-2 also interacted with LPS containing H type I under weakly acidic conditions. Furthermore, Gal-3 is structurally more stable than Gal-2 and interacted with not only H type I but also Lewis X. These results suggest that Gal-2 and -3 function cooperatively in the defense against *H. pylori* in the stomach under different pH conditions.

## Figures and Tables

**Table 1:** Analysis of the composition of lipopolysaccharide extracted from *Helicobacter pylori.* Chemical composition of the lipopolysaccharides isolated from *H*. *pylori* ATCC43504, purified by DNase, RNase, and proteinase K treatments, followed by ultracentrifugation. The molar ratio of _L_-*glycero*-_D_-*manno*-Heptose was 2.0.

Components	% (*w*/*w*)	nmol/mg	Molar Ratio
Sugars			
Fucose	3.0	181	3.2
Ribose	8.6	572	10.1
Galactose	6.4	355	6.3
Glucose	4.2	234	4.1
_D_-g*lycero*-_D_-*manno*-Heptose	3.8	179	3.2
_L_-g*lycero*-_D_-*manno*-Heptose	2.4	113	2.0
*N*-Acetylglucosamine	7.3	332	5.9
Phosphorous			
Total	3.3	333	5.9
Fatty acids			
Palmitic acid	0.5	20	0.4
Octadecanoic acid	3.2	113	2.0
3-Hydroxy-palmitic acid	0.9	35	0.6
3-Hydroxy-octadecanoic acid	4.5	149	2.6

## Data Availability

The raw data supporting the conclusions of this article will be made available by the authors on request.

## Supplementary Materials

The following supporting information can be downloaded at https://www.mdpi.com/article/10.3390/ijms25168725/s1.
