# Supplementary material for: Galectin-2 Agglutinates Helicobacter pylori via Lipopolysaccharide Containing H Type I Under Weakly Acidic Conditions
Source: Int J Mol Sci. 2024 Aug 10;25(16):8725. doi: 10.3390/ijms25168725 (PMC11354322; doi:10.3390/ijms25168725)
Supplement: Supplementary file 1 [file ijms-25-08725-s001.zip › ijms-3121945-supplementary.pdf]

Supplementary Figure 1

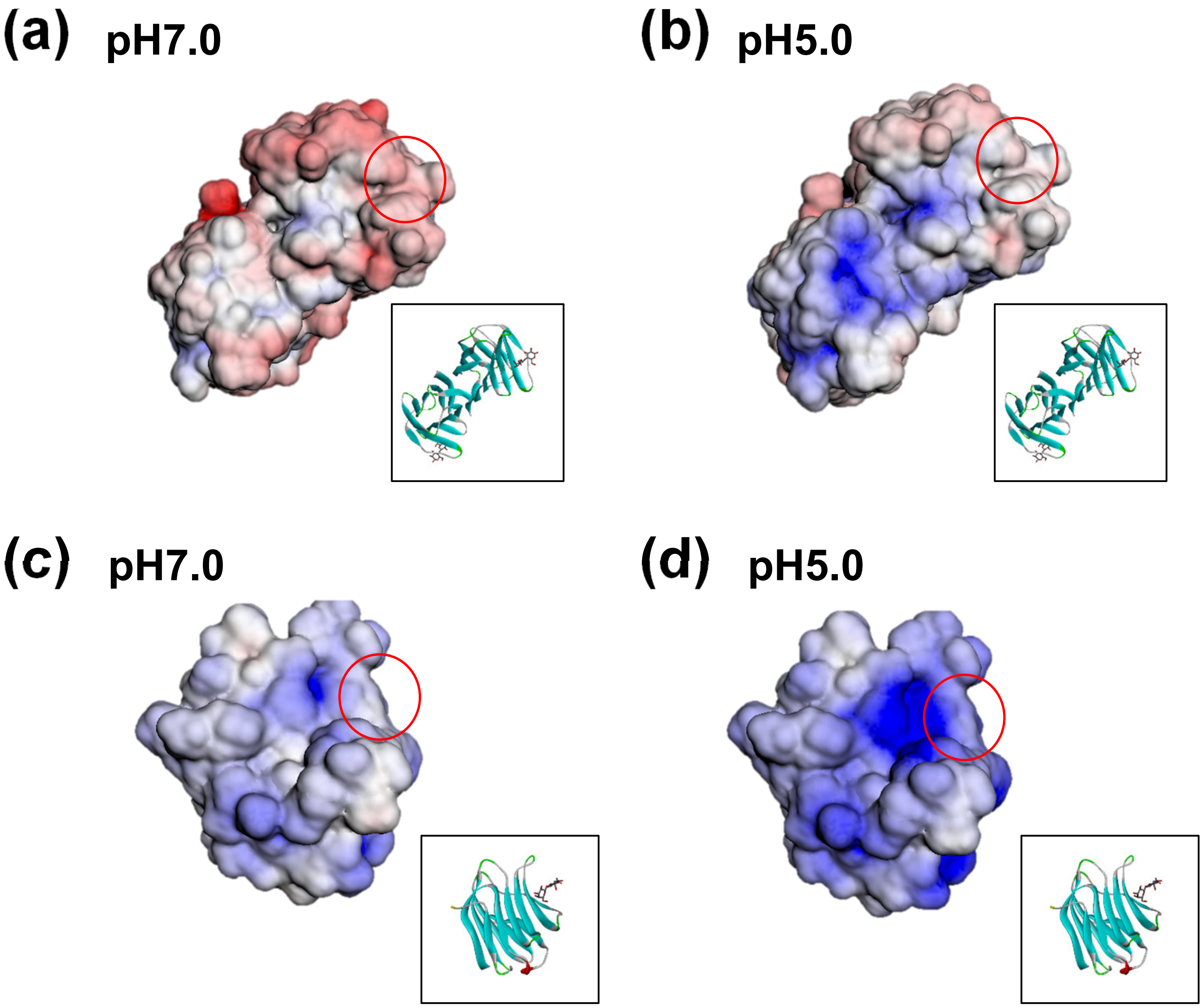

Supplementary Figure 1. Comparisons of molecular surface of hGal-2(as a dimer) and hGal-3 (as a monomer).

Electrostatic potential maps of hGal-2 at pH 7.0 (a) and 5.0 (b), and hGal-3 at pH 7.0 (c) and 5.0 (d) were calculated using the Poisson-Boltzmann server ver. 3.6.2 (https://server.poissonboltzmann.org/). Red and blue indicate negative and positive charge, respectively. At the right end of each figure, Figure 2a is displayed in (a) and (b), Figure 4a is displayed in (c) and (d) for reference. Red circles indicate the sites to which lactose binds.

Supplementary Figure 2

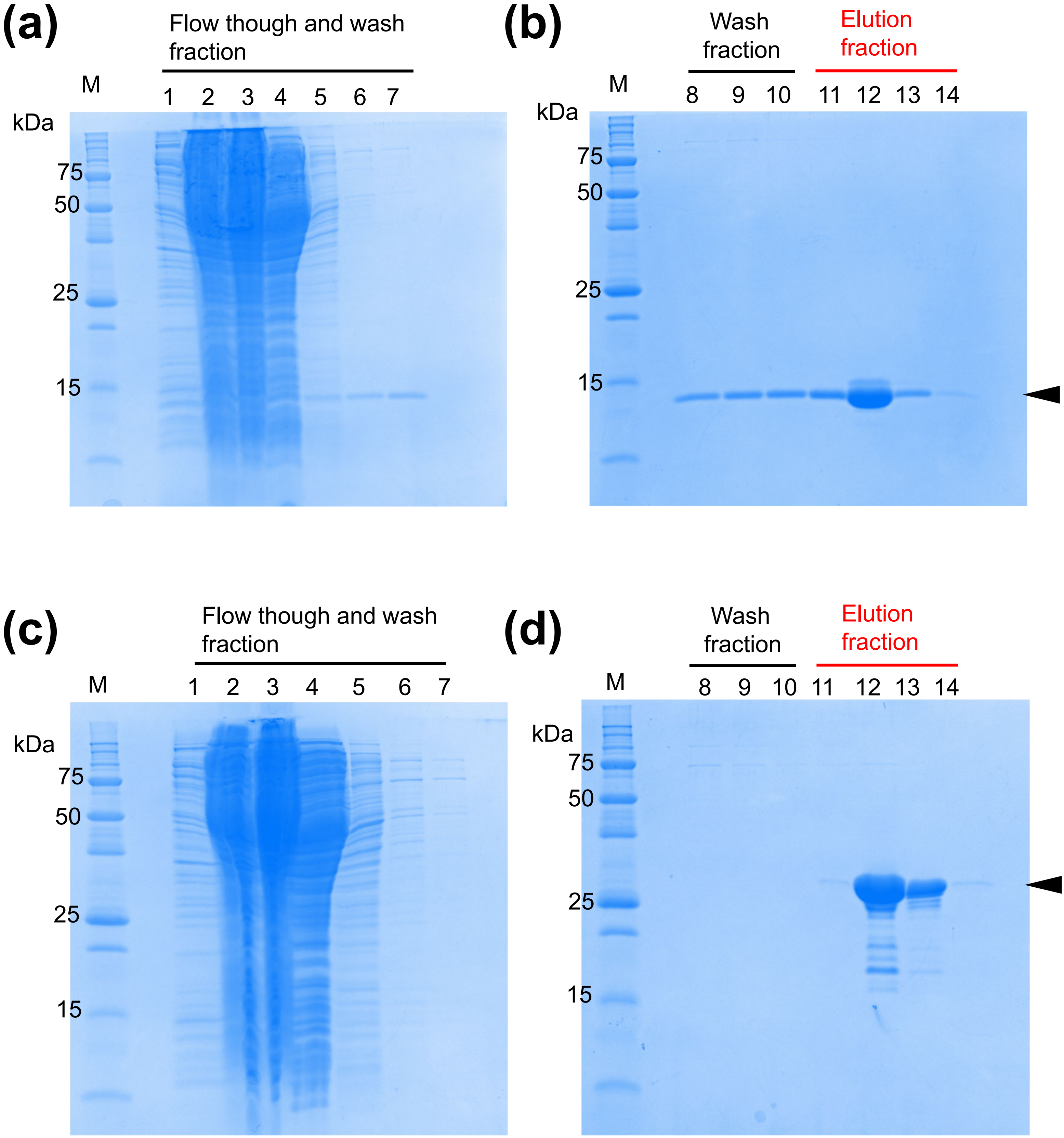

Supplementary Figure 2. Coomassie Brilliant Blue staining of collected fraction after affinity chromatography of hGal-2 and hGal-3.

Collected fractions after affinity chromatography using an asialofetuin-immobilized column were separated by SDS-PAGE. Then the gels (hGal-2: a and b, hGal-3: c and d) were stained using Coomassie G-250 stain (Bio-Rad, Hercules, CA, USA). The molecular weight of hGal-2 and hGal-3 are 14 and 32 kDa, respectively. Arrowheads indicate the position of hGal-2 or hGal-3. M, molecular mass markers.
